# Causal effects of psoriasis on breast cancer risk: The contribution of emotional distress explored by Mendelian randomization

**DOI:** 10.1097/MD.0000000000049330

**Published:** 2026-06-12

**Authors:** Lu Ye, Hui Yang, Danyan Qian, Zhongyuan Lu

**Affiliations:** a Outpatient Department, The 903rd Hospital of the Joint Logistic Support Force, Hangzhou, Zhejiang, China.

**Keywords:** breast cancer, mediation analysis, Mendelian randomization, negative emotions, psoriasis

## Abstract

Psoriasis is a chronic inflammatory disease associated with an increased risk of various cancers, including breast cancer. However, the mechanisms underlying this association remain poorly understood. Negative emotional states, commonly experienced by individuals with psoriasis, may play a role in mediating this link. This study aims to investigate the causal effect of psoriasis on breast cancer risk and explore the potential mediating role of negative emotions using Mendelian randomization (MR) approaches. Genetic instruments for psoriasis and negative emotions were sourced from large-scale genome-wide association studies. Univariate MR was first performed to evaluate the direct impact of psoriasis and its subtypes on breast cancer risk. Subsequently, multivariable MR (MVMR) was used to account for the combined effects of psoriasis and negative emotions. Mediation analysis was conducted to assess the extent to which negative emotions mediate the relationship between psoriasis and breast cancer. Sensitivity analyses, including tests for heterogeneity and horizontal pleiotropy, were performed to validate the robustness of the results. The univariate MR analysis demonstrated a significant positive association between psoriasis and the risk of breast cancer [overall psoriasis: odds ratio (OR) = 1.022, 95% confidence interval (CI): 1.005–1.039, *P* = .013; psoriasis vulgaris: OR = 1.020, 95% CI: 1.004–1.036, *P* = .013; guttate psoriasis: OR = 1.017, 95% CI: 1.005–1.030, *P* = .006; psoriatic arthropathies: OR = 1.018, 95% CI: 1.002–1.035, *P* = .032; other and unspecified psoriasis: OR = 1.017, 95% CI: 1.001–1.034, *P* = .035]. In the multivariable MR analysis, after adjusting for negative emotions, the association between psoriasis and breast cancer became nonsignificant (overall psoriasis: OR = 1.012, 95% CI: 0.988–1.037, *P* = .333), indicating that negative emotions may not significantly mediate this relationship. Mediation analysis revealed that worry and anxiety feelings accounted for a significant portion of the effect of psoriasis on breast cancer risk (*P* = .017). These findings suggest that worry and anxiety may serve as mediators in the causal pathway linking psoriasis to an increased risk of breast cancer. This emphasizes the importance of addressing emotional health in individuals with psoriasis, which may have implications for cancer prevention strategies. Further research into the underlying biological mechanisms is warranted to better understand the clinical implications of these results.

## 1. Introduction

Psoriasis is a chronic, immune-mediated inflammatory skin disorder that affects a significant portion of the global population.^[1–3]^ The condition is characterized by hyperproliferation of keratinocytes, leading to the formation of plaques, typically on the scalp, elbows, and knees.^[4,5]^ While psoriasis primarily affects the skin, it is also associated with a wide range of comorbidities, including cardiovascular diseases,^[6,7]^ metabolic disorders,^[8,9]^ and certain types of cancer.^[10]^ Among these, psoriasis has been suggested to increase the risk of malignancies, including nonmelanoma skin cancer, lymphoma, and breast cancer. However, the underlying mechanisms linking psoriasis to cancer risk, particularly breast cancer, remain poorly understood.

Breast cancer is one of the most common cancers worldwide, with millions of new cases diagnosed each year.^[11,12]^ The development of breast cancer is a complex, multifactorial process that involves genetic, hormonal, and environmental factors.^[13–15]^ Genetic factors are one of the main causes, particularly mutations in genes such as breast cancer susceptibility gene 1 and breast cancer susceptibility gene 2,^[16]^ which increase the risk of breast cancer by affecting deoxyribonucleic acid repair mechanisms. Hormonal factors also play a significant role, especially the stimulating effect of estrogen on breast cell proliferation.^[17]^ Prolonged exposure to hormones can significantly increase the risk of breast cancer. In terms of molecular characteristics, breast cancer can be classified into different subtypes, such as estrogen receptor-positive, human epidermal growth factor receptor 2-positive, and triple-negative breast cancer, each with different pathogenic mechanisms. Additionally, the tumor microenvironment, including immune cells and fibroblasts, also contributes to the growth and metastasis of the tumor. Chronic inflammation is a key factor in the development of breast cancer, with inflammatory mediators activating signaling pathways that promote cancer cell proliferation and resistance.^[18]^ Finally, lifestyle and environmental factors, such as smoking, obesity, and radiation exposure, are known risk factors.^[19]^ Psoriasis may influence the risk of breast cancer through various pathways, including immune dysregulation,^[20]^ chronic inflammation,^[21]^ and systemic cytokine imbalance.^[22]^ The chronic inflammatory state in psoriasis results in the upregulation of pro-inflammatory cytokines, such as tumor necrosis factor-alpha, interleukin-6, and interleukin-17, which are known to promote tumorigenesis. Furthermore, the systemic nature of psoriasis can lead to dysregulated immune surveillance, which may contribute to increased cancer susceptibility. Despite these plausible biological mechanisms, the direct causal relationship between psoriasis and breast cancer risk remains unclear.

Negative emotional states, including depression and anxiety, are frequently observed in individuals with psoriasis,^[23,24]^ given the chronic nature of the disease and its impact on quality of life. These negative emotions are thought to exacerbate the inflammatory processes that underpin psoriasis and may also contribute to increased cancer risk.^[25,26]^ The biological mechanisms through which negative emotions influence cancer risk include dysregulation of the hypothalamic-pituitary-adrenal (HPA) axis,^[27]^ increased production of cortisol, and heightened levels of inflammatory mediators.^[28]^ The HPA axis is a key pathway linking psychological stress, neuroendocrine regulation, and breast cancer progression. Chronic stress can persistently activate the HPA axis and elevate glucocorticoid levels, thereby suppressing antitumor immunity, altering the inflammatory microenvironment, and promoting tumor progression.^[29]^ Meanwhile, emerging evidence indicates that breast tumors themselves can reciprocally disrupt HPA axis rhythmicity and negative feedback regulation, forming a bidirectional tumor–brain–immune regulatory loop,^[30]^ suggesting that neuroendocrine regulation may represent a potential therapeutic target in breast cancer. The question arises whether these negative emotional states might mediate the effect of psoriasis on breast cancer risk, potentially acting as a key link in the causal pathway.

Mendelian randomization (MR) is a powerful tool that can help clarify causal relationships between risk factors and outcomes by utilizing genetic variants as instrumental variables (IVs).^[31–33]^ This study aims to apply MR to investigate the causal effect of psoriasis on breast cancer risk, with a specific focus on the potential mediating role of negative emotions. By leveraging large-scale genetic data, this study will provide novel insights into the biological mechanisms that underlie the psoriasis-breast cancer association and identify possible targets for intervention. Understanding the role of negative emotions as a mediator in this pathway could open avenues for more holistic approaches to the prevention and management of both psoriasis and breast cancer, improving the overall health outcomes of affected individuals.

## 2. Materials and methods

### 2.1. Study design

**Figure 1.**
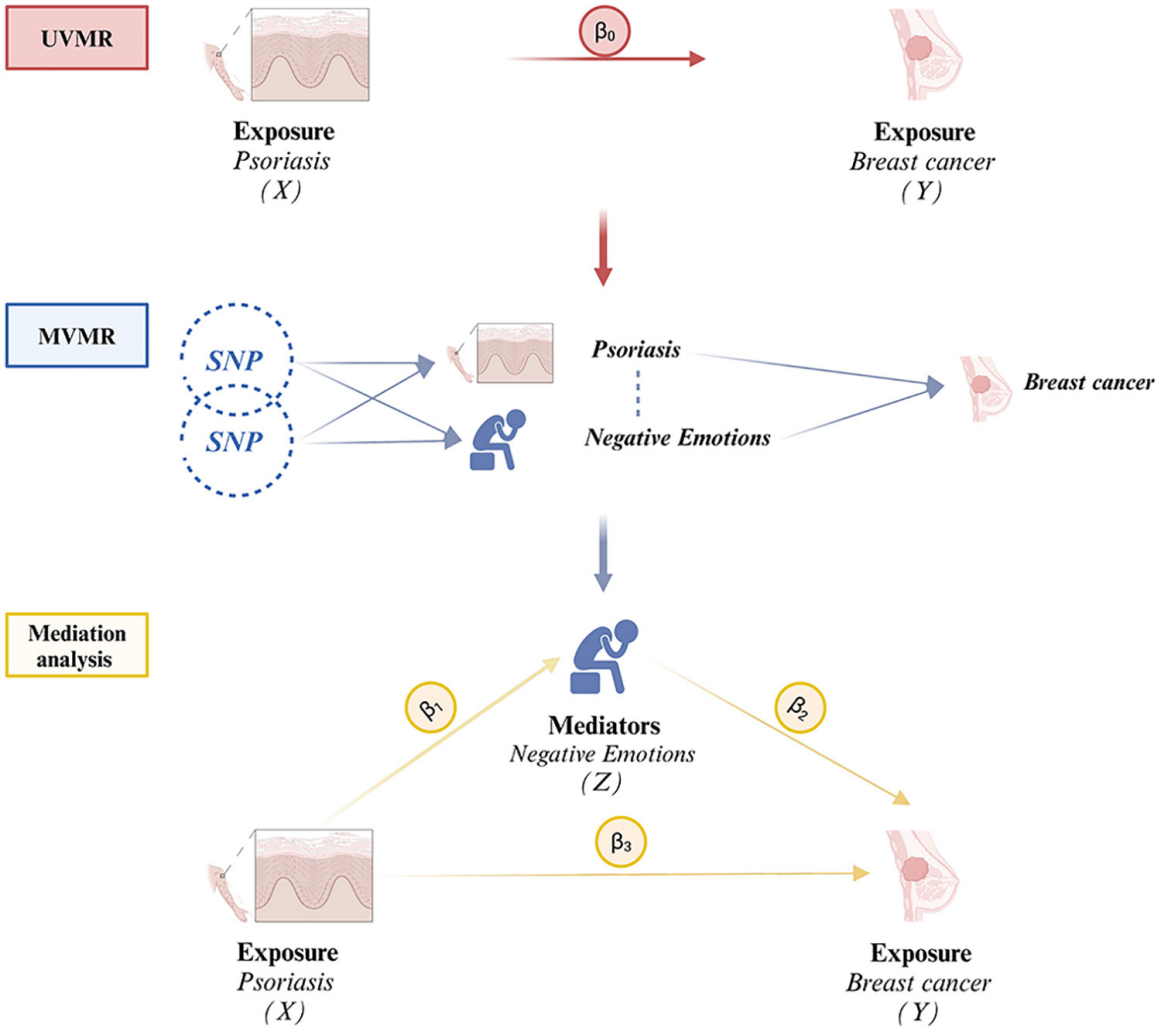
Flowchart illustrating the design and analytical process of this MR study. MR = Mendelian randomization, MVMR = multivariable Mendelian randomization, SNP = single nucleotide polymorphism, UVMR = univariate Mendelian randomization.

This study aimed to explore the causal link between psoriasis, negative emotions, and breast cancer risk through MR analysis. To assess the direct impact of psoriasis on breast cancer risk, univariate MR (UVMR) was initially conducted. Considering a previous meta-analysis that included over 100,000 participants, which showed that negative emotions significantly increased the risk of breast cancer by nearly 60%,^[34]^ multivariable MR (MVMR) was subsequently applied to evaluate the combined effects of psoriasis and negative emotions.^[35]^ Additionally, mediation analysis was performed to examine the role of negative emotions in mediating the relationship between psoriasis and breast cancer risk^[36]^ (Fig. 1).

### 2.2. Data resource

The genome-wide association studies summary data on psoriasis and its subtypes were obtained from the latest R12 release of the FinnGen database (https://www.finngen.fi/en) (“finngen_R12_L12_PSORIASIS” for psoriasis, case = 12,760; “finngen_R12_L12_PSORI_VULG” for psoriasis vulgaris, case = 7912; “finngen_R12_L12_PSORI_GUTTATE” for guttate psoriasis, case = 496; “finngen_R12_M13_PSORIARTH” for psoriatic arthropathies, case = 4290; “finngen_R12_L12_PSORI_NAS” for other and unspecified psoriasis, case = 2452), which is a large-scale research initiative that combines genetic data with health records from Finnish individuals to investigate the genetic risk factors of a variety of diseases.^[37]^ The database provides valuable insights into the genetic basis of diseases prevalent in the Finnish population and is widely used for identifying genetic associations and improving healthcare strategies. Additionally, the large genome-wide association studies summary data on breast cancer and negative emotions were sourced from the Integrative Epidemiology Unit database (https://gwas.mrcieu.ac.uk/) (“ebi-a-GCST004988” for breast cancer, case = 76,192; “ukb-b-6519” for worrier/ anxious feelings, case = 255,812; “ebi-a-GCST005902” for depression, case = 113,769), which contains diverse publicly available genetic datasets, facilitating research on the genetic foundations of numerous traits and diseases^[38]^ (Table 1). The data on negative emotions were independent of the breast cancer population to avoid any sample overlap.

**Table 1:** Summary of GWAS datasets utilized in this MR study.

Type	ID of GWAS	Trait	Case	Population	Year
Exposure	finngen_R12_L12_PSORIASIS	Psoriasis	12,760	European	2024
Exposure	finngen_R12_L12_PSORI_VULG	Psoriasis vulgaris	7912	European	2024
Exposure	finngen_R12_L12_PSORI_GUTTATE	Guttate psoriasis	496	European	2024
Exposure	finngen_R12_M13_PSORIARTH	Psoriatic arthropathies	4290	European	2024
Exposure	finngen_R12_L12_PSORI_NAS	Other and unspecified psoriasis	2452	European	2024
Mediator	ukb-b-6519	Worrier/anxious feelings	255,812	European	2018
Mediator	ebi-a-GCST005902	Depression	113,769	European	2018
Outcome	ebi-a-GCST004988	Breast cancer	76,192	European	2017

GWAS = Genome-wide association study, MR = Mendelian randomization.

### 2.3. Selection of IVs

**Figure 2.**
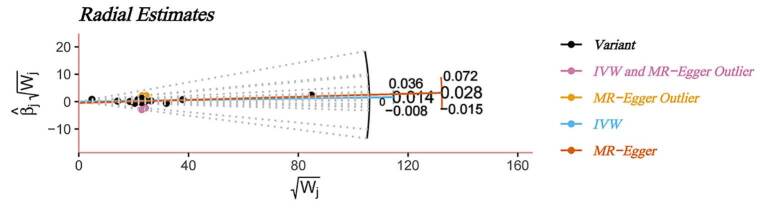
Radial MR plot identifying outlier SNPs. IVW = inverse-variance weighted, MR = Mendelian randomization, SNPs = single nucleotide polymorphisms.

In this study, single nucleotide polymorphisms (SNPs) were selected as IVs for psoriasis and its subtypes in UVMR, since the distribution of most SNPs is relatively uniform in European populations. To meet the core assumptions of an MR study, the selection criteria for SNPs were set with a *P* value threshold of < 5 × 10^−8,[39]^ meaning SNPs were chosen based on this critical threshold. Additionally, to avoid the impact of linkage disequilibrium (LD), SNPs with an *R*^2^ < 0.001 were selected,^[40]^ and the length of the LD block was restricted to within 10,000 kb to minimize the influence of LD. To prevent weak instrument bias, SNPs with an *F*-statistic > 10 were defined as free from weak instrument bias (*F*=*β*^2^/SE^2^).^[41]^ It is worth noting that in both the UVMR and mediation analysis, the same stringent selection criteria were applied to negative emotions (*P* value threshold = 5 × 10^−8^, *R*^2^ < 0.001, *F*-statistic > 10). Finally, radial MR was employed to remove outlier SNPs (Fig. 2). After the rigorous filtering process, the remaining SNPs were selected as genetic proxies for subsequent MR analysis.

### 2.4. MR analysis

In this study, the inverse-variance weighted method (IVW) was selected as the primary UVMR analysis approach.^[42]^ Considering potential heterogeneity and horizontal pleiotropy, we additionally employed 3 MR analysis methods: MR-Egger,^[43]^ weighted median^[44]^ and weighted mode.^[45]^ In addition to UVMR, MVMR analysis was further performed to determine whether psoriasis serves as an independent risk factor for breast cancer. Finally, mediation analysis was conducted to assess the role of negative emotions in mediating the effect of psoriasis on breast cancer. Sensitivity analyses were conducted using various methods. Firstly, Cochran *Q* test was employed to assess heterogeneity. In the presence of heterogeneity, the IVW results were analyzed using the random effects model; in the absence of heterogeneity, the fixed effects model was used. Additionally, the MR-Egger intercept test was applied to assess horizontal pleiotropy. A *P* value < .05 was considered statistically significant.

## 3. Results

### 3.1. Genetic effects of psoriasis and its subtypes on breast cancer in UVMR

**Figure 3.**
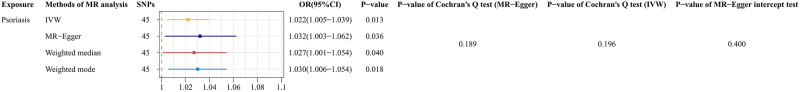
Causal effect of overall psoriasis on breast cancer in UVMR. CI = confidence interval, IVW = inverse-variance weighted, MR = Mendelian randomization, OR = odds ratio, SNP = single nucleotide polymorphism, UVMR = univariate Mendelian randomization.

**Figure 4.**
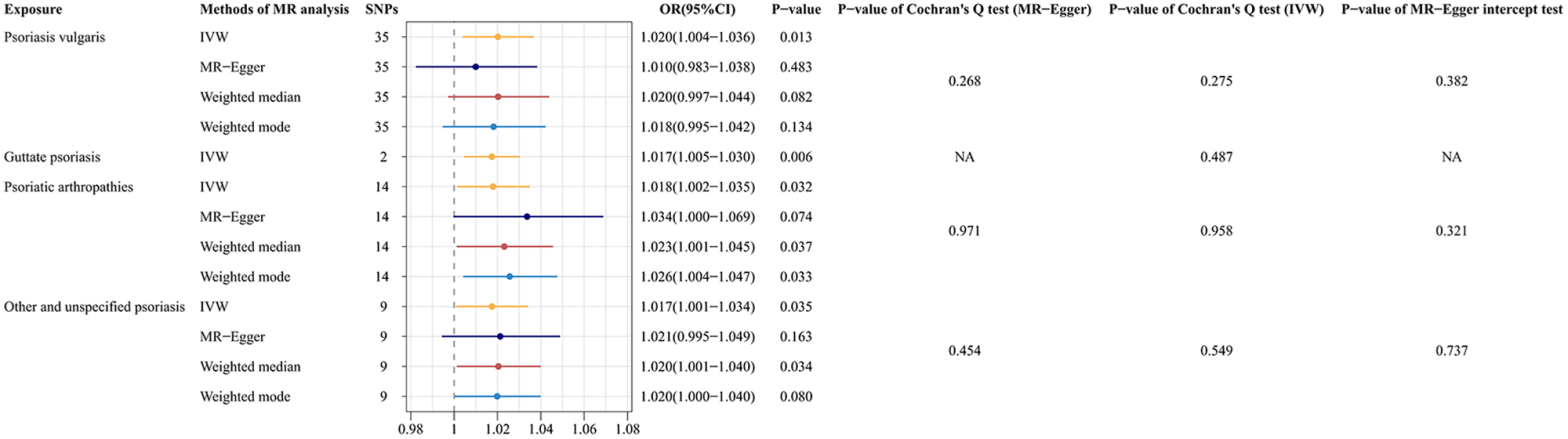
Causal effect of psoriasis subtypes on breast cancer in UVMR. CI = confidence interval, IVW = inverse-variance weighted, MR = Mendelian randomization, OR = odds ratio, SNP = single nucleotide polymorphism, UVMR = univariate Mendelian randomization.

According to the results obtained through the IVW model, the overall condition of psoriasis, along with its subtypes, is significantly related to breast cancer [overall psoriasis: odds ratio (OR) = 1.022, 95% confidence interval (CI): 1.005–1.039, *P* = .013; psoriasis vulgaris: OR = 1.020, 95% CI: 1.004–1.036, *P* = .013; guttate psoriasis: OR = 1.017, 95% CI: 1.005–1.030, *P* = .006; psoriatic arthropathies: OR = 1.018, 95% CI:1.002–1.035, *P* = .032; other and unspecified psoriasis: OR = 1.017, 95% CI: 1.001–1.034, *P* = .035]. Both Cochran *Q* test (*P* > .05) and the MR-Egger intercept (*P* > .05) failed to detect any evidence of significant heterogeneity or horizontal pleiotropy (Figs. 3 and 4).

### 3.2. Genetic effects of psoriasis on breast cancer in MVMR

**Figure 5.**
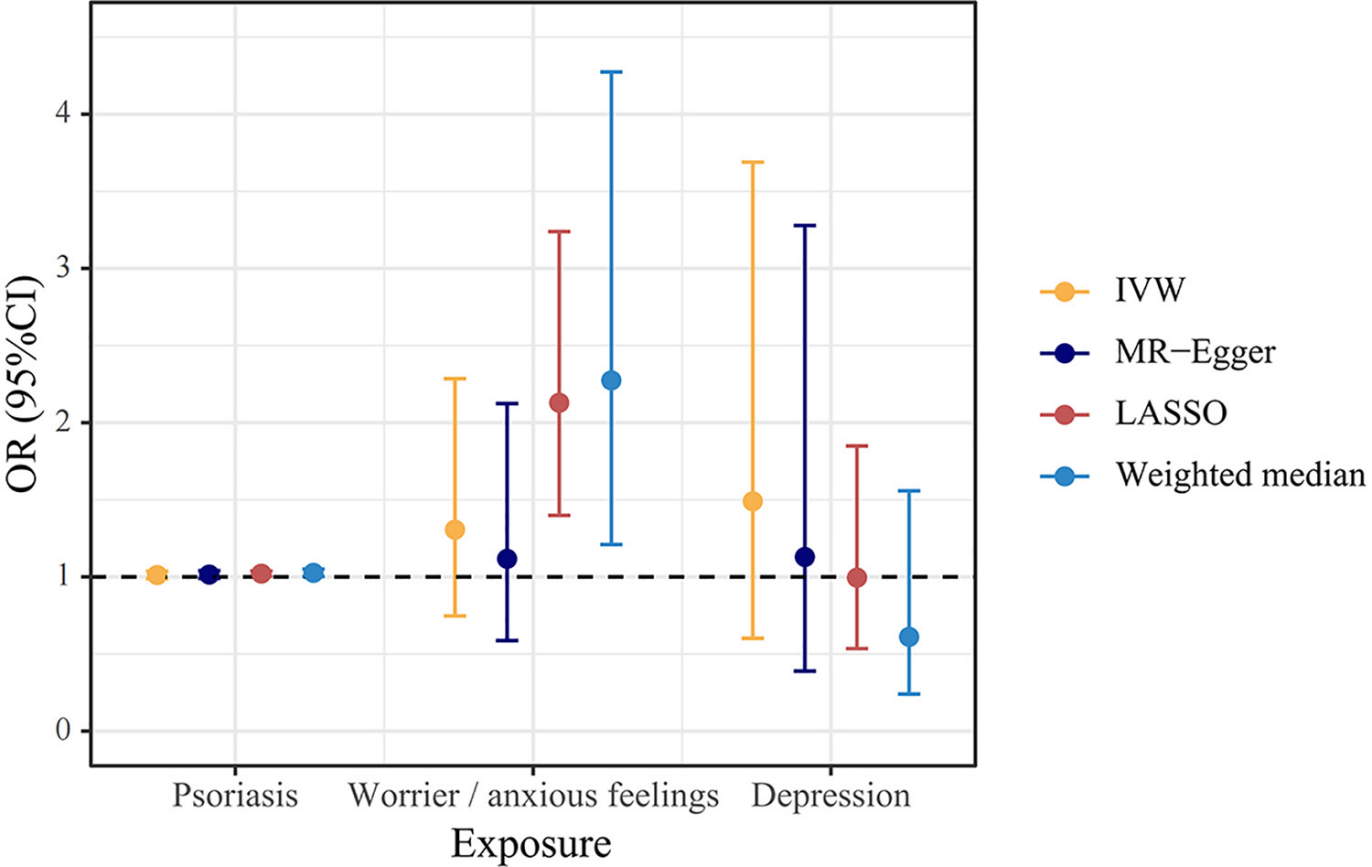
Causal effect of overall psoriasis on breast cancer in MVMR. CI = confidence interval, IVW = inverse-variance weighted, MR = Mendelian randomization, MVMR = multivariate Mendelian randomization, OR = odds ratio, SNP = single nucleotide polymorphism.

After adjusting for negative emotions, including worry/anxious feelings and depression, the causal effect of psoriasis on breast cancer became nonsignificant (overall psoriasis: OR = 1.012, 95% CI: 0.988–1.037, *P* = .333). This suggests that negative emotions play a role in mediating the relationship between psoriasis and breast cancer (Fig. 5).

### 3.3. The role of negative emotions in mediating psoriasis and breast cancer risk

**Figure 6.**
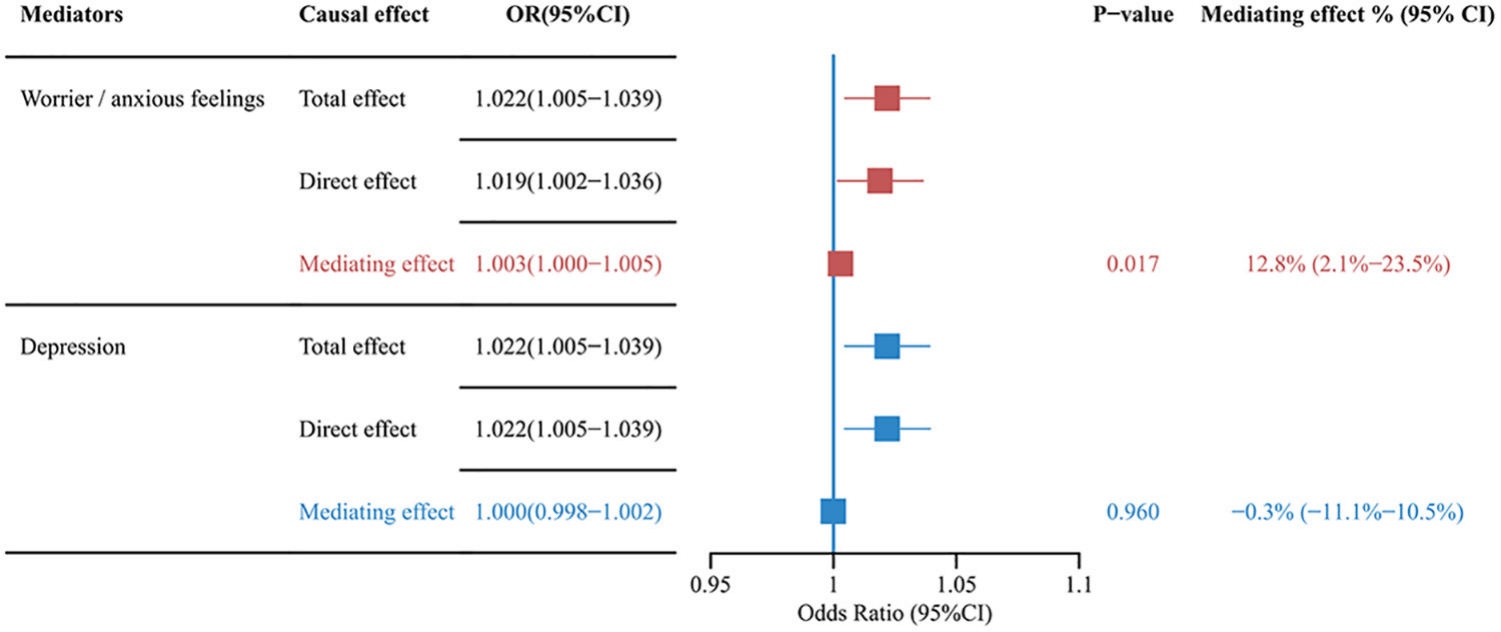
The role of negative emotions in mediating psoriasis and breast cancer risk. CI = confidence interval, OR = odds ratio.

Further mediation analysis revealed that worry/anxious feelings significantly mediate the relationship between psoriasis and breast cancer (*P* = .017), while depression does not appear to play a mediating role (*P*=.960) (Fig. 6).

## 4. Discussion

Our study aimed to investigate the causal relationship between psoriasis and breast cancer, focusing on the potential mediating role of negative emotions. Psoriasis is a chronic inflammatory skin disease that has been associated with systemic inflammation and various extra-cutaneous manifestations.^[46]^ Given the growing evidence linking chronic inflammation to cancer development, we hypothesized that psoriasis might increase the risk of breast cancer. Our findings support this hypothesis, demonstrating a significant causal relationship between several psoriasis subtypes and breast cancer. Additionally, we found that worry/anxious feelings partially mediate this relationship, suggesting that emotional stress may play a role in the development of breast cancer in individuals with psoriasis.

The association between chronic inflammation and cancer has been well documented,^[47]^ and it is increasingly understood that conditions like psoriasis, which are marked by persistent inflammation, could contribute to carcinogenesis. In the case of psoriasis, this inflammatory state is driven by the dysregulation of immune responses, particularly T-cell activation and cytokine production,^[48,49]^ which could influence systemic processes beyond the skin. These immune mediators are known to promote tumorigenesis by creating a pro-inflammatory microenvironment,^[50]^ which may foster the development of cancers, including breast cancer. Furthermore, psoriasis is often accompanied by comorbidities, such as metabolic syndrome,^[51]^ which are also known to increase the risk of breast cancer. The inflammatory burden, therefore, likely contributes to an elevated risk of developing breast cancer in individuals with psoriasis, supporting our study’s conclusions.

In addition to the direct inflammatory effects, our study revealed the mediating role of negative emotions, specifically worry and anxiety, in the relationship between psoriasis and breast cancer. Worry and anxiety are common psychological responses in individuals with chronic diseases like psoriasis,^[52]^ which often involve visible symptoms and could severely impact a person’s quality of life. Psychological stress has been linked to increased levels of pro-inflammatory cytokines,^[53]^ which can exacerbate the inflammatory processes associated with psoriasis. Moreover, chronic stress has been shown to impair immune function and may create an environment conducive to cancer development.^[54]^ The finding that negative emotions mediate the relationship between psoriasis and breast cancer suggests that managing psychological distress may be an important component of mitigating the cancer risk in psoriasis patients.

MR was employed in this study to assess the causal relationship between psoriasis and breast cancer, which strengthens the validity of our findings. MR is a powerful technique that uses genetic variants as IVs to infer causal relationships between exposures and outcomes, minimizing the confounding and reverse causality often present in observational studies.^[55,56]^ By leveraging genetic data, MR allows for a more robust analysis of the relationship between psoriasis and breast cancer, as genetic variants are less susceptible to biases like reverse causality or measurement error. In addition, MR offers the advantage of reducing the confounding effect of environmental factors, such as smoking or diet, that might otherwise influence the observed relationship. The inclusion of mediation analysis further allowed us to assess the role of negative emotions, providing a more comprehensive view of the mechanisms linking psoriasis to breast cancer.

However, there are some limitations to consider.^[57]^ While MR is a powerful tool, it assumes that the genetic variants used as instruments only influence the outcome through the exposure of interest, a condition known as the exclusion restriction assumption. If this assumption is violated, the results may be biased. In addition, MR cannot fully account for gene-environment interactions or other complex biological processes that might influence the relationship between psoriasis and breast cancer. Furthermore, the mediation analysis, while informative, cannot establish a definitive causal link between negative emotions and breast cancer, as it is limited by the available genetic data and the complexity of psychological factors. Additionally, the genetic instruments used for negative emotions, such as anxiety or worry, may not fully capture the nuanced aspects of emotional distress that contribute to cancer risk.

## 5. Conclusion

In conclusion, our study provides compelling evidence of a significant causal relationship between psoriasis and breast cancer, with negative emotions, particularly worry and anxiety, serving as mediators in this association. These findings highlight the importance of addressing both the inflammatory and psychological aspects of psoriasis management. A multidisciplinary approach that integrates medical and psychological interventions is essential to reduce cancer risk and improve overall quality of life. Future research should explore the molecular mechanisms underlying the interaction between inflammation, psychological distress, and cancer development, and work towards developing comprehensive interventions.

## Author contributions

**Conceptualization:** Zhongyuan Lu.

**Investigation:** Hui Yang.

**Methodology:** Hui Yang.

**Visualization:** Danyan Qian.

**Writing – original draft:** Lu Ye.
